# E-MOVIE - Experimental MOVies for Induction of Emotions in neuroscience: An innovative film database with normative data and sex differences

**DOI:** 10.1371/journal.pone.0223124

**Published:** 2019-10-03

**Authors:** Antonio Maffei, Alessandro Angrilli

**Affiliations:** 1 Department of General Psychology, University of Padova, Padova, Italy; 2 CNR Neuroscience Institute, Section of Padova, Padova, Italy; 3 PNC–Padova Neuroscience Center, University of Padova, Padova, Italy; Universita degli Studi di Pisa, ITALY

## Abstract

The need for a validated set of emotional clips to elicit emotions in more ecological experiments is increasing. Here we present the validation of a new database of emotional films, named E-MOVIE, which includes, in this first validation phase, 39 excerpts arranged in six categories, three negative (Fear, Sadness and Compassion), two positive (Erotic and Scenery) and a Neutral category. Notably, Compassion and Scenery are new in the field as they were not included in other databases. The clips in E-MOVIE are characterized by homogenous durations of approximately two minutes, which make them suitable for psychophysiological research. In order to study the affective profile prompted by each category 174 participants (112 women) rated the movies on multiple dimensions, namely valence and arousal, intensity and discreteness of the induction of one of the six basic emotions and, finally, intensity of the experience of the emotional states defined by a series of emotional adjectives. Erotic clips were effective in the elicitation of a positive emotional state, characterized by high levels of arousal and excitement. On the other hand, Fear clips (selected without blood to avoid disgust reaction) prompted an affect characterized by high arousal, low valence and high levels of reported fear and anxiety. Women reported greater unpleasantness, distress, anxiety and jittery than men to the three negative categories. Compassion clips, characterized by the depiction of crying characters, were able to induce an affective state dominated by sadness and feeling touched, consistent with an empathic reaction to emotional sufferance. Sadness clips, instead, elicited an affective state characterized by sadness together with distress and angst. We also demonstrated that clips depicting natural environments (i.e. Scenery) prompted in the viewer a surprised, inspired affective state, characterized by high valence and arousal (especially in males), a result which suggests that their past categorization as neutral stimuli was inaccurate and problematic.

## Introduction

The induction of an emotional state in the lab represents a long-standing issue in the investigations of human emotions [1]. A critical aspect, faced by experimenters in this field, concerns the need to find a balance between the psychometric control of the main perceptual variables (brightness, color, sound level, sound tone, duration, etc.), and the ability to induce an effective, ecological and spontaneous emotion in the lab.

There are many different methods available for emotion induction, each with advantages and limitations, and these are: emotional imagery, presentation of visual stimuli (slides and videos), presentation of sound or music, recollection of autobiographical memories and, more recently, virtual reality [2–6].

Affective pictures are one of the most widely used tool, comprising many important characteristics that made its usage widespread. Pictures are indeed a class of stimuli very easy to implement in an experiment, providing the experimenter with the flexibility to switch between different presentation paradigms. They can be used in a great variety of experimental designs, from simple passive viewing to more complex designs, in which slide presentation is mixed with other tasks. Moreover, they put low demand to participants, making easy their use with different populations (i.e. children, clinical samples). Another useful feature of pictures is that they provide to experimenters the ability of controlling the perceptual characteristics of the stimuli, and even to experimentally manipulate them [7], thus allowing a great overall control. Finally, it is important to acknowledge that the great diffusion of pictures as a mean of emotion elicitation is due to the great availability of validated databases of pictorial stimuli, such as the universally acknowledged International Affective Pictures System [4], POFA [8], KDEF [9], NAPS [10].

Nevertheless, images have some important limitations that can be surpassed by other methods. One of the most critical shortcomings of pictures is that they are static, therefore the affective experience prompted by picture viewing is neither strong, nor ecological enough compared to that induced with a dynamic modality, such as video presentation [11].

Indeed, film clips allow a multimodal stimulation of the viewer, with a simultaneous and coherent engagement of both the visual and the auditory sensory systems. In the context of mood induction, they are suitable for elicitation of basic emotions, like Fear or Disgust, as well as for the induction of more complex feelings [1,12]. Furthermore, the recent diffusion of modern video editing software applications has made very easy to select and edit video clips for experimental use.

Two seminal works in this field have been done by Philippot [13] and Gross and Levenson [14], who first systematically investigated the effectiveness of movie excerpts for emotional induction and built a validated dataset of stimuli. Their works have been followed in recent years by new efforts for providing extended, wider, and updated datasets of short movies [1,12,15–19]. Each of this dataset has distinct strengths, aiming at filling the gap in the availability of standardized stimuli, which has limited the application of this method. The present work sought to contribute to this process, by focusing on those aspects in emotional film validation that, so far, received less attention. Moreover, it aimed at providing a database of film clips explicitly designed to meet the needs of neuroimaging and psychophysiological research, while retaining the flexibility of those databases not developed to meet the constraints of neuroscience research. The first use of emotional movies to study the physiological correlates of emotions dates back to several decades ago [20], but recent years have seen a surge in the use of this kind of stimuli [21–23], due to the unique advantages that they provide. Nevertheless, most of past and current studies are still carried out with a few clips lacking large validation data and this might limit the generalizability of the findings.

### The present research

A review of the existing libraries of film clips shows that there is a wide variability in the duration of the stimuli included, with clips lasting less than a minute [18,24] to clips with durations of over seven minutes [12]. What is more surprising is that this variability is large within most of databases [12,14,25]. This inconsistency is due to the fact that different films are able to elicit the same target affect in different ways (i.e. different plots, different scenes, different soundtracks), leaving researchers with stimuli which vary in their duration. Moreover, some emotions could arise from very brief stimulation, like Disgust or Fear, while secondary, complex emotions, characterized by lower biological relevance, need more time to develop (e.g. disappointment). Although this is a minor issue for those studies based on self-report evaluation only, large variations between the experimental stimuli may lead to a problematic interpretation of the results, and this is particularly true in the field of psychophysiology and neuroscience (in which having stimuli with equal or, at least, comparable durations represents an essential requirement).

For what concerns the optimal duration of an emotional stimulus there are no clues in the literature, and past authors did not explicitly address this issue when building their sets. Since there is no clear criterion for deciding how long should last a stimulation for eliciting an emotion, most authors of past database used, as proper duration, the integrity of the scene, a choice which led to the quoted large variability of film duration within many databases. Here we decided to include only clips with completed scenes of approximately two minutes duration, according to a theoretical perspective that views the emotional experience as a phasic phenomenon superimposed to a tonic affective state. Such duration should be long enough to provide the viewer with an understanding of the plot, leading to a coherent and stable change of his/her affective state. Shorter clips (i.e. one minute or less) may not be long enough to induce a complex emotion, engage the attention of the viewer and making possible the process of “willing suspension of disbelief” [1,26], which is critical for the ecology of the emotional experience achieved through film watching. On the other hand, using longer clips (i.e. three minutes or more) may increase the difficulty to disengage from the stimulus, thus prompting carryover effects on the following excerpts within the experimental paradigm. In addition, using longer excerpts could potentially impact the number of stimuli employed in an experimental paradigm with multiple clips, hence reducing the generalizability of the results.

Another critical issue addressed in the present work regards the film categories that should be included and their target affective states. Most of the available databases generally include stimuli selected from thriller and horror movies in order to elicit Fear and Disgust, covering the spectrum of the negative affect. On the other hand, less agreement has been found in the selection of film categories for the elicitation of positive emotions. The general approach adopted so far was the inclusion of clips extracted from film comedies and/or stand-up comedy, in order to target positive affective states most commonly reported as Joy, Happiness and Amusement. This selection strategy of existing database has been developed within the theoretical framework of the “basic emotion theory” [27]. Nevertheless, there are some exceptions like the work of Samson and colleagues [18] which relied on a simpler classification among positive, negative and mixed emotional states, and the work of Carvalho and colleagues [24] which was based on a dimensional view of emotions.

In the present research, we sought to build a dataset including film categories able to cover the spectrum of both positive and negative affective states and to introduce some new categories that have rarely or never been considered. In general, very homogenous categories have been developed to avoid cross-contamination among different emotions underlying confounded/borderline categories (see e.g. below past research on Fear often confounded with Disgust).

Concerning the range of negative emotions, we decided to include the categories of Fear, Sadness and Compassion. In the past, clips selected to elicit Fear consisted of scenes characterized by tense and threatening situations that are often accompanied by the depiction of violence, blood and/or mutilation. Although these clips are very powerful in eliciting an affective reaction in the viewer, there is evidence that stimuli characterized by the depiction of blood and wounds elicit a large overlapping between the feelings of Fear and Disgust [12], thus reducing the homogeneity of the selected category. Moreover, it was repeatedly proven that stimuli depicting blood and mutilation represent a category which prompt very distinctive physiological reactions [28,29], therefore including clips featuring these kind of elements could lead to confounded emotions and, eventually, bias the results. Thus, we decided to focus exclusively on clips able to elicit feelings of fear and anxiety related to the anticipation of a threat, without the depiction of violence and blood, and aiming at inducing a “pure” fear experience.

Sadness is another category that has consistently been included in previous validations, usually comprising clips depicting scenes of grief and loss, whose typical feature is the representation of one or more crying characters. Here we aimed to operate a finer distinction within the broad category of sadness-eliciting clips, by retaining in the Sadness category clips featuring themes of loneliness and helplessness, while the selection of excerpts featuring scenes of characters crying for a loss or a separation to a novel category, was defined Compassion. Crying is a biologically grounded behavior evolved to communicate sufferance to others, seeking for help and prompting a motivated approach [30–32], therefore clips depicting crying scenes were expected to evoke feelings of sadness but also a stronger empathic response [33], which also determines feeling of compassion, empathic concern and prosocial tendency. Instead, clips featuring scenes of loneliness, without showing any crying character, were expected to prompt a sad emotional state, characterized also by angst and distress, in which empathy for others and its associated prosocial motivation was expected to be less prominent in guiding the viewer’s emotional reaction. Indeed, this distinction is founded on recent neurobiological evidences [34] suggesting that although empathy is a fundamental mechanism guiding reaction toward another person sufferance (either physical or emotional), two different emotional states might arise from it: one involving a prosocial motivation (Compassion) and one which is more self-centered and characterized by increased distress. Within this framework, we hypothesized that the presence within a sad scene of a powerful biosocial cue like crying would shift the balance toward a compassionate focused-to-others sadness, while the absence of crying display would trigger a self-focused emotional state of sadness characterized by distress. In terms of motivational disposition, the Sadness clips would evoke a (weak) tendency to withdraw the situation, while the Compassion content would induce a tendency to approach the crying person to relieve her sufferance.

For what concerns the elicitation of positive emotions, we decided to include the Erotic and Scenery categories. Stimuli depicting erotic scenes have been largely used in past research, with both pictures and films, as a powerful source of pleasant and high arousal emotional state, that could be considered as a complement to the affect elicited by threatening stimuli [35–41]. Interestingly, while erotic clips have been widely used in past isolated studies on emotions as single non validated strong positive stimulations, previous standardized and validated datasets of film clips for emotional induction largely neglected this category of contents, with the exception of the work of Carvahlo and colleagues [24].

Unlike erotic scenes, clips depicting natural landscapes (we termed them “Scenery”) have been already included in other databases [1,24], but they have been used as clips inducing a Neutral condition. Here we argue against this definition and use, after analyzing the growing body of research [42–46] that showed that exposure to natural environments and landscapes, both direct and indirect (like exposure trough pictures and videos), has a positive effect on mood, emotional well-being and facilitates recovery form stressful events. Therefore, in the light of past evidence, using these stimuli as affectively neutral category seems inappropriate. They should rather be considered as elicitors of positive affect. Starting with the mentioned analysis of past literature and dataset we have developed and validated a first set of 39 clips divided into six homogenous emotional categories.

## Method

### Participants

One hundred and seventy-four students from University of Padova (112 females and 62 males) were enrolled in this study, in different phases and classes, in exchange of credit course or monetary reward (13 €). Mean age of the sample was 21.3 years (s.d. = 2.6 years). The investigation was approved by the local Ethics Committee (Comitato Etico della Ricerca Psicologica Area 17, University of Padova), has been conducted according to the principles expressed in the Declaration of Helsinki and all the participants gave their written consent to participate in the study.

### Stimuli

Thirty-nine excerpts from commercial motion pictures have been selected and edited for being included in our validation sample according to several criteria: 1) lasting approximately two minutes, 2) providing a consistent and easily to understand development of the plot (without sudden changes or transitions), 3) showing the most arousing part of the clip in the second minute, 4) to form highly homogenous categories of clips. The clips were a-priori divided into six categories of interest, thus the sample was arranged as showed in Table 1.

**Table 1:** List of film clips with corresponding mean and standard deviation for valence and arousal, divided by gender. See Supplementary Material for clips normative ratings for all the items considered in the emotional assessment.

FILM	CATEGORY	VALENCE	AROUSAL
Lust [1]	*EROTIC*	M = 6 (1.4) F = 5.3 (1.7)	M = 5.2 (2) F = 5.6 (2.3)
Lust [2]	*EROTIC*	M = 5.7 (1.7) F = 4.8 (2)	M = 5.7 (1.8) F = 5.6 (2.1)
Monster’s Ball	*EROTIC*	M = 6.8 (1.3) F = 5.7 (1.7)	M = 6.1 (2) F = 5.7 (2.2)
The Notebook	*EROTIC*	M = 6.3 (1.2) F = 6.7 (1.6)	M = 5.2 (1.7) F = 6.2 (1.7)
Underworld	*EROTIC*	M = 6.4 (1) F = 6.2 (1.4)	M = 4.6 (2) F = 5.1 (2.2)
Supernatural	*EROTIC*	M = 6 (1.2) F = 6.2 (1.3)	M = 4.1 (1.9) F = 5.1 (2)
40 Days and 40 Nights	*EROTIC*	M = 6 (1.7) F = 6.4 (1.6)	M = 4.5 (1.9) F = 5.2 (2)
Bbc’s Planet Earth: Seasonal Forests	*SCENERY*	M = 6.5 (1.6) F = 6.3 (1.4)	M = 4.5 (2.3) F = 3.4 (2.4)
Bbc’s Planet Earth: Deserts	*SCENERY*	M = 6.2 (1.6) F = 5.8 (1.4)	M = 4.9 (2.4) F = 4.2 (2.5)
Bbc’s Planet Earth: Fresh Water	*SCENERY*	M = 6.7 (1.5) F = 6.7 (1.5)	M = 4.9 (2.5) F = 4.3 (2.4)
Bbc’s Planet Earth: Mountains	*SCENERY*	M = 6.6 (1.7) F = 6.1 (1.6)	M = 5.1 (2.2) F = 3.9 (2.6)
SPACE	*SCENERY*	M = 6.5 (1.7) F = 6.4 (1.5)	M = 4.8 (2.6) F = 4.4 (2.5)
Bbc’s Great Barrier Reef	*SCENERY*	M = 6.9 (1.7) F = 7.2 (1.5)	M = 4.5 (2.5) F = 4.4 (2.3)
The Road	*SADNESS*	M = 3.7 (1.8) F = 2.8 (1.6)	M = 4.8 (2.1) F = 4.3 (2.3)
Blood Diamond	*SADNESS*	M = 4.9 (1.6) F = 4.1 (1.6)	M = 4.2 (2.1) F = 4.8 (2.1)
K-19	*SADNESS*	M = 3.7 (1.3) F = 3.3 (1.4)	M = 4.3 (2) F = 4.2 (2.2)
Million Dollar Baby [1]	*SADNESS*	M = 3.4 (1.5) F = 2.7 (1.6)	M = 4.3 (2.1) F = 4.7 (2.5)
Million Dollar Baby [2]	*SADNESS*	M = 3.1 (1.3) F = 2.2 (1.3)	M = 4.7 (2.1) F = 5.1 (2.4)
The Hours	*SADNESS*	M = 2.6 (1.7) F = 3.5 (1.9)	M = 5.7 (2) F = 6.8 (2)
The Champ	*COMPASSION*	M = 3.2 (1.6) F = 2.3 (1.5)	M = 4.4 (2.1) F = 5.1 (2.1)
My Girl	*COMPASSION*	M = 3.4 (1.4) F = 2.2 (1.2)	M = 3.9 (2.1) F = 5.3 (2.3)
Lost	*COMPASSION*	M = 3.4 (1.7) F = 2.7 (1.8)	M = 4.6 (2.1) F = 5.2 (2.2)
Pearl Harbor	*COMPASSION*	M = 3.4 (1.6) F = 2.5 (1.5)	M = 4.8 (2.2) F = 4.7 (2.3)
Armageddon	*COMPASSION*	M = 4.1 (2) F = 3.5 (2)	M = 5.3 (2.2) F = 5.4 (2.3)
The Pursuit Of Happiness	*COMPASSION*	M = 3.2 (1.6) F = 3.3 (2.1)	M = 5.1 (2.1) F = 5.6 (2.3)
The Others	*FEAR*	M = 4.4 (1.8) F = 3.4 (1.4)	M = 4.9 (2.1) F = 5.7 (2.2)
The Sixth Sense	*FEAR*	M = 3.3 (1.8) F = 2.2 (1.4)	M = 5.4 (2) F = 6 (2.3)
Deep Red [1]	*FEAR*	M = 4 (1.6) F = 3.2 (1.6)	M = 4.5 (2.3) F = 5.5 (2.4)
Deep Red [2]	*FEAR*	M = 4.2 (1.7) F = 3.8 (1.5)	M = 4.4 (2.1) F = 4.1 (2.5)
Gothika	*FEAR*	M = 3.3 (1.5) F = 2.5 (1.5)	M = 5.3 (2) F = 6.2 (2.2)
The Silence of The Lambs	*FEAR*	M = 3.7 (1.4) F = 2.8 (1.4)	M = 4.7 (2.1) F = 5.4 (2.4)
Vacancy	*FEAR*	M = 3.7 (1.8) F = 2.6 (1.7)	M = 5.3 (2.2) F = 6.7 (2.3)
Globe Trekker’s London City Guide	*NEUTRAL*	M = 5.3 (1.3) F = 5.3 (1.4)	M = 3.3 (1.9) F = 2.9 (2.1)
Globe Trekker’s New York City Guide	*NEUTRAL*	M = 5.1 (1.2) F = 5.4 (1.4)	M = 3 (2) F = 2.7 (2.3)
Globe Trekker’s Paris City Guide	*NEUTRAL*	M = 5.1 (1.2) F = 5.3 (1.2)	M = 2.6 (1.8) F = 2.6 (2.1)
Italian Documentary: Bronte	*NEUTRAL*	M = 4.5 (1.6) F = 4.9 (1.3)	M = 2 (1.3) F = 2 (1.7)
Italian Documentary: Pietraperzia	*NEUTRAL*	M = 4.7 (1.3) F = 5 (1.2)	M = 2 (1.5) F = 1.9 (1.6)
Italian Documentary: Calamonaci	*NEUTRAL*	M = 4.6 (1.8) F = 4.7 (1.3)	M = 2.2 (1.7) F = 2 (1.6)
Italian Documentary: Quartesolo	*NEUTRAL*	M = 4.5 (1.5) F = 5 (1.2)	M = 1.9 (1.3) F = 2.1 (1.7)

All the selected clips were presented with their original audio in Italian language, with the exception of Scenery clips which were presented with a musical soundtrack. It is worth to acknowledge that, although some of the clips here included have been a-priori selected without looking at past literature, a few of them resulted to be already present in other databases [12,14,24]. However most of the clips of E-MOVIE are new in the literature, especially the excerpts included in the Erotic and Scenery categories. The clips were selected from commercial movies with an available English audio version, in order to make easy for English-speaking researchers to re-edit in English the clips included in E-MOVIE. Currently, it is possible to download the Italian version of the clips used in this study from the Open Science Framework (*https*:*//osf*.*io/scrbp/*?*view_only=2b3081d3e1a44852a8a0f23ff5f396f7*).

A single, continuous, clip was obtained after editing the excerpts with Adobe Premiere CS5, with a final resolution of 1280x720 pixels. Within the final clip, the order of the excerpts was pseudo-randomized, namely there were no two consecutive excerpts belonging to the same category.

### Procedure

Participants were tested in groups in different session, with each session comprising a group of no more than 40 participants. Participants were separated with an empty seat in order not to invade the privacy of the neighbors. Furthermore, males and females were tested separately, in order to avoid socially related confounds, especially due to the sensitive nature of some clips (i.e. the Erotic category) in which gender differences are known [47,48].

Each session started with an introductory clip, that comprised four excerpts (not included in the experimental sample) resembling the categories under investigation. The first clips served to make the participants familiar with both the kind of movies they were going to watch and the questionnaire they were requested to complete after each clip. At the end of the trial phase, the experimental clips were presented as a continuous randomized stream of clips with 20 s intervals for the evaluation. Each session lasted for approximately two and a half hours.

### Emotional assessment

After each clip, participants were required to fill an inventory which included several analogue and likert scales, selected in order to cover a broad spectrum of information about their emotional experience. The inventory was divided in three sections: the first included a paper-and-pencil version of the *Self-Assessment Manikin* [49], in which participants were asked to rate the pleasantness of the emotional state elicited by the clip and the arousal felt during the viewing. The second section consisted of a Basic Emotions evaluation, in which participants were presented with a list of six basic emotions (Fear, Sadness, Rage, Disgust, Joy and Surprise) plus a Neutral item and they were asked to rate the degree of each emotion felt on a 9-points Likert scale. In the third section of the inventory, participants were asked to evaluate the clip using a list of 11 emotional adjectives, selected in order to encompass a broad span of positive and negative feelings, and to indicate on a 5 point Likert Scale how much of the listed Emotional Adjectives they fitted with. In addition, participants were also requested to indicate if they had previously watched the clip (Familiarity score).

### Statistical analysis

Subjects’ responses were collapsed for each film category and were analyzed with a series of repeated-measures ANOVAs. For the analysis of SAM ratings, ANOVAs were designed with one within-subjects factor (Film category) and one between-subjects factor (Sex). For the analysis of Basic Emotions we were interested in modeling both the ability of each film category to elicit the target basic emotion (efficacy) as well as to measure the intensity of the target emotion with respect to that induced by the non-target emotions (discreteness). Therefore, we designed an ANOVA with two within-subjects factor (Film category and Emotion), and one between-subjects factor (Sex) to model gender differences. For the Emotional Adjectives, we carried out a series of ANOVAs (separately for each adjective) with one within-subjects factor (Film Category) and one between-subjects factor (Sex), in order to model the efficacy of each film category to elicit the target affective state. In addition, we computed (separately for each film category) a series of ANOVAs with one within-subjects factor (Emotional Adjectives) and one between-subjects factor (Sex), in order to model for each category the affective state that was elicited with greater intensity. Due to problems in data collection, sample size for three emotional adjectives (Sad, Anxious and Enchanted) and for the Basic Emotion evaluation was 134 (79 women). When appropriate, the reported p-values are corrected with the Greenhouse-Geisser procedure. Significant effects were further explored using Newman-Keuls post-hoc test with a significance level set at *p*<0.05.

In order to analyze the impact of Familiarity (i.e. having seen previously the clip) on perceived Valence and Arousal, we carried out a series of Welch’s t-test [50] on valence and arousal ratings using familiarity as a predictor. In order to handle the large difference found on familiarity score for the clips, we tested only a subgroup of clips which have been seen previously by at least 15 participants. With this criterion, 19 clips surpassed the Familiarity threshold and a statistical test was computed on Valence and Arousal ratings by comparing participants who found the clip familiar with those who did not.

In the Supplemental Material (S1 File), the mean ratings separately for genders, for each film and emotional scale are reported.

## Results

### SAM

**Fig 1:**
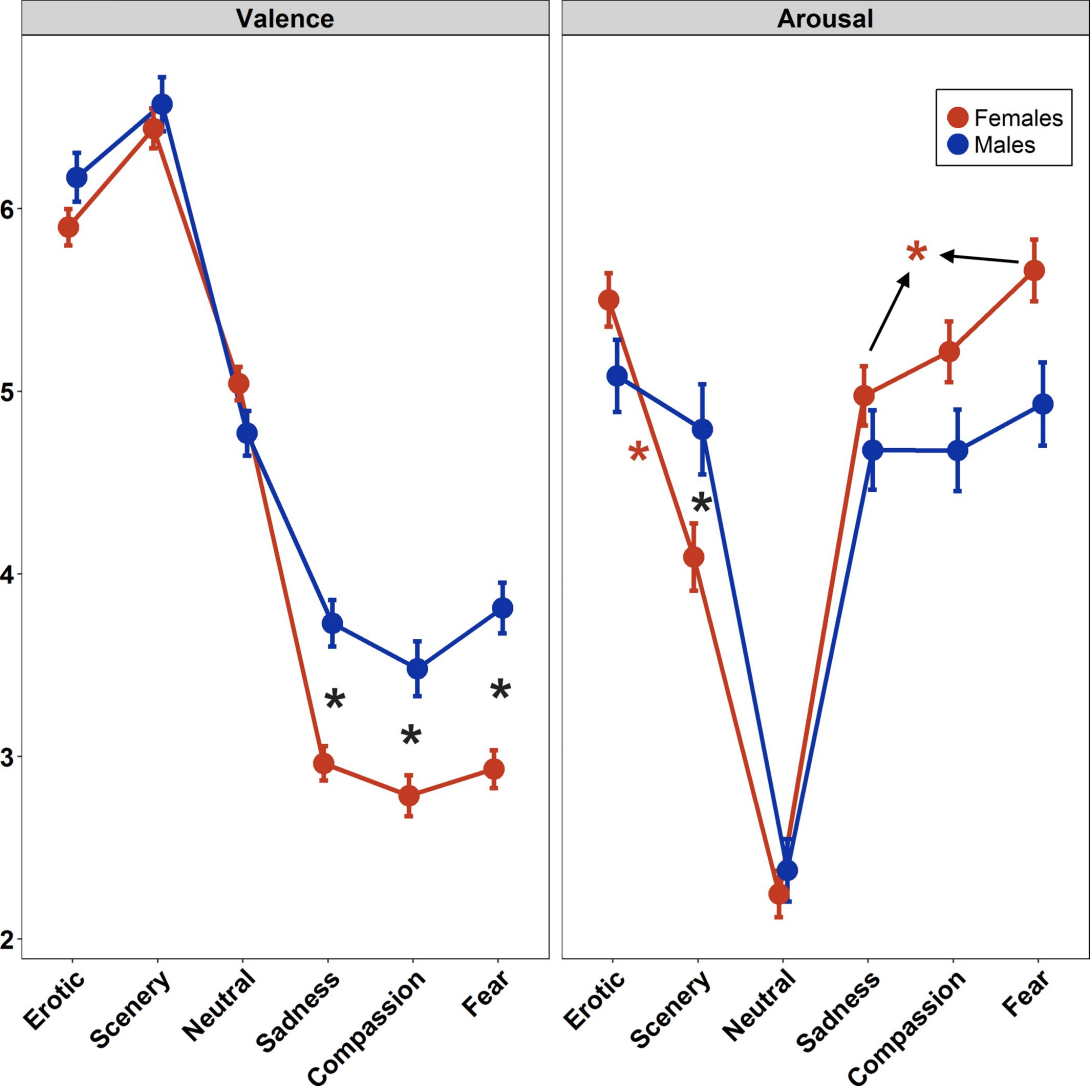
Effect of film categories on self-reported valence and arousal. Asterisks indicate significant (p< 0.05) post-hoc effect. Bars represent Standard Error (SE).

ANOVA on Valence revealed both Film Category (*F*(5,860) = 357.97, *p*<0.0001, η^2^_p_ = 0.67) and Sex (*F*(1,172) = 19.45, *p*<0.0001, η^2^_p_ = 0.10) significant main effects (Fig 1, left panel). Furthermore, also the interaction of the two factors was significant (*F*(5,860) = 8.08, *p*<0.0001, η^2^_p_ = 0.04). Post-hoc analysis of the interaction term revealed significant differences, for both males and female participants, among Erotic, Scenery and Neutral clips and between these categories and the three negative ones (Sadness, Compassion and Fear), which instead were not differentiated by the valence judgments. In both genders, Scenery clips were perceived as more pleasant than the Erotic ones. Furthermore, gender differences were found between judgments on the three negative film categories, with women reporting lower Valence (more unpleasantness) during the viewing of these films compared to men.

ANOVA on the Arousal ratings showed a significant effect of Film Category (*F*(5,860) = 135.87, *p*<0.0001, η^2^_p_ = 0.44) and a significant Film Category*Sex interaction (*F*(5,860) = 7.34, *p*<0.0001, η^2^_p_ = 0.04). Post-hoc test on the Film main effect showed that Erotic and Fear clips scored highest while Neutral clips scored lowest on arousal ratings (Fig 1, right panel). Post-hoc analysis of the interaction revealed a different pattern between the sexes, with men reporting the same significant higher level of arousal for all the affective categories compared with the Neutral one, while women, during Erotic and Fear excerpts, experienced higher arousal levels with respect to Scenery, Neutral and Sadness clips. Also Scenery and Sadness elicited higher arousal than Neutral category. Finally, men judged Scenery clips as more arousing than women did.

**Fig 2:**
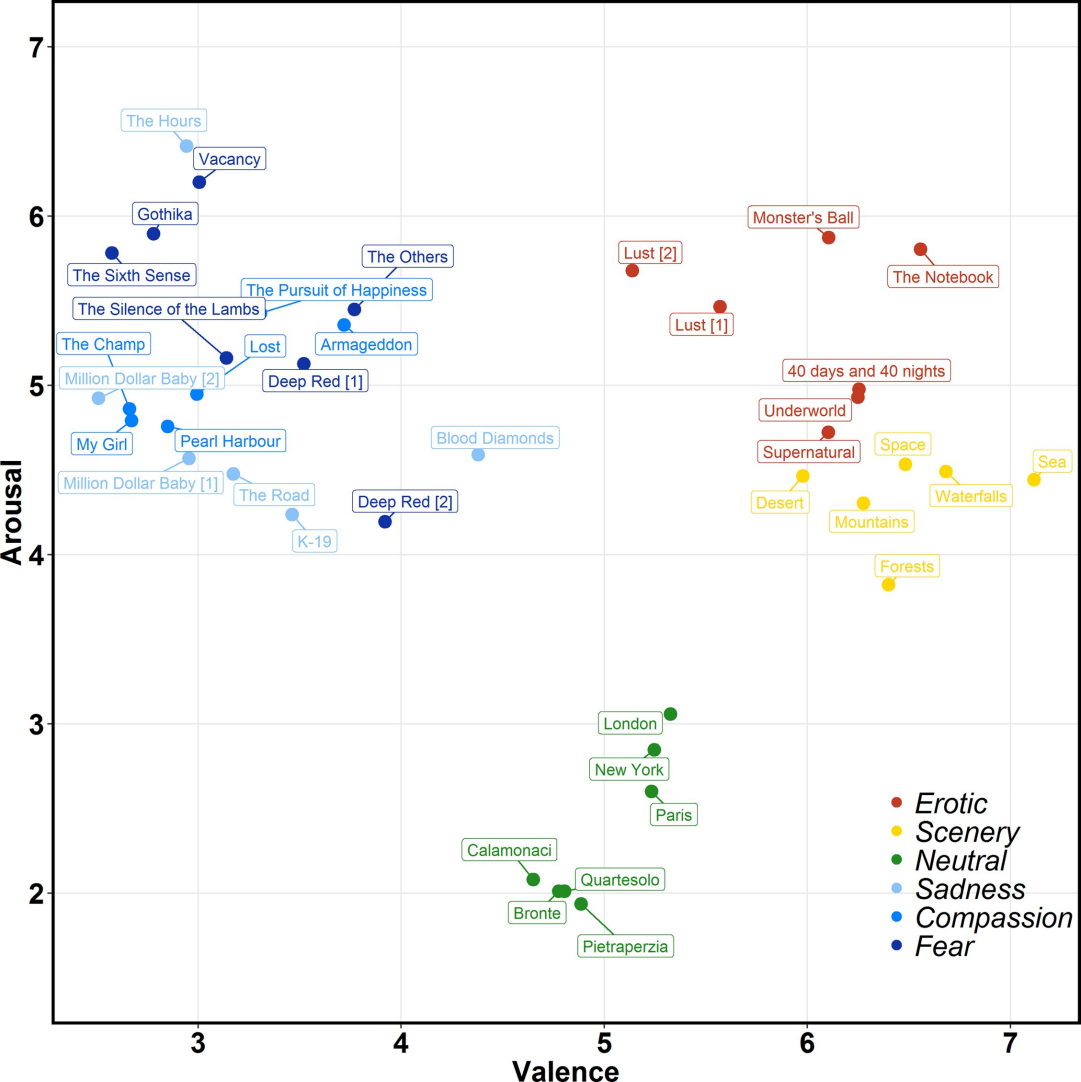
Distribution of the 39 film clips in the valence-arousal affective space.

The combination of Valence and Arousal of each film in a bi-dimensional affective space is shown in Fig 2.

### Basic emotions

**Fig 3:**
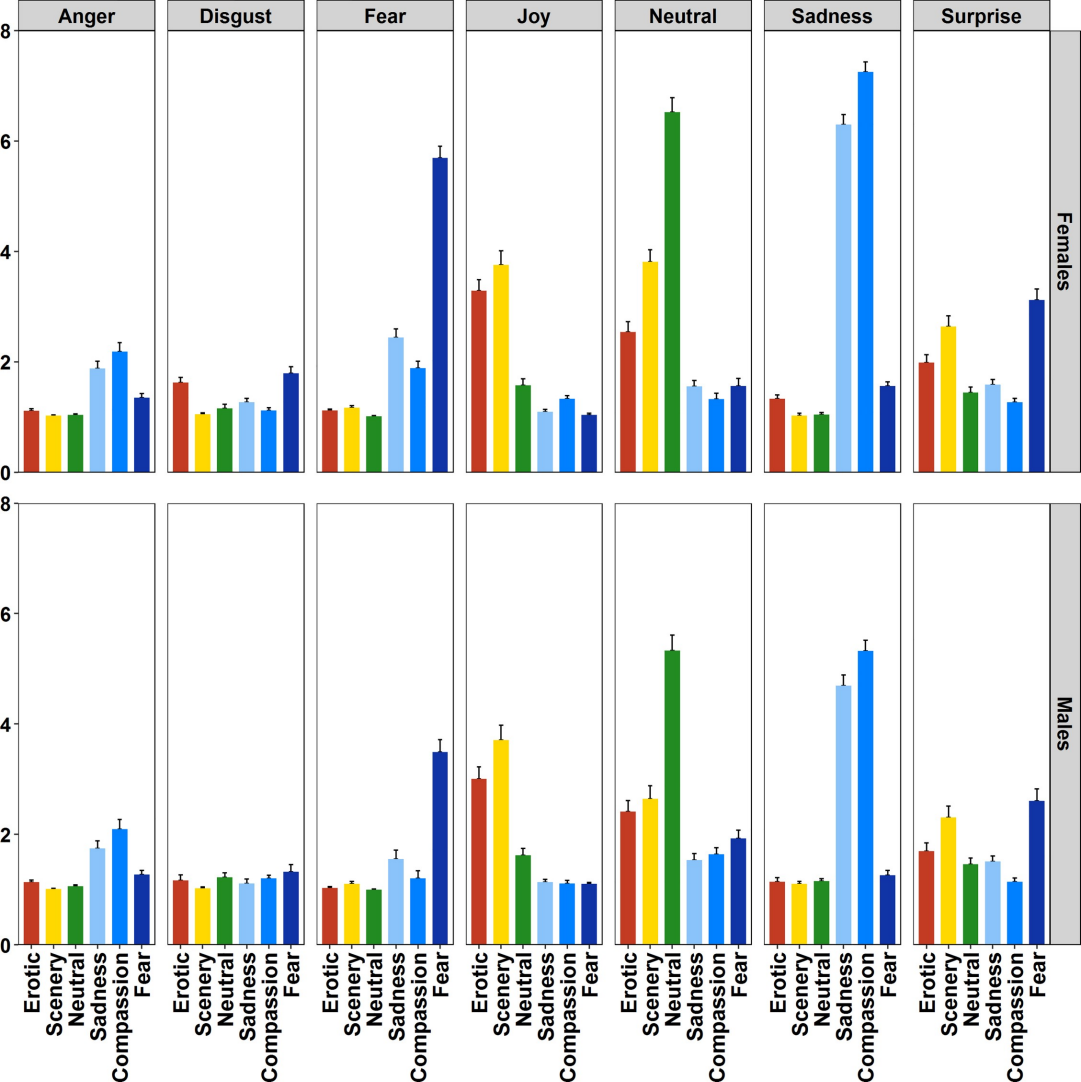
Evaluation of the 6 film categories according to the main seven basic emotions. Bars represent SE.

ANOVA carried out on Basic Emotion ratings found the Film Category*Emotion (*F*(30,3990) = 285.45, *p*<0.0001, η^2^_p_ = 0.68) and the Film Category*Emotion*Sex, *F*(30,3990) = 11.43, *p*<0.0001, η^2^_p_ = 0.07) significant interactions. Post-hoc analysis computed on the Film Category*Emotion*Sex interaction revealed several results on the attribution of basic emotions to the six Film categories. Participants experienced low levels of Anger for the whole sample of clips, as they reported low ratings of anger to all categories compared to other emotions, similar in men and women (Fig 3, first panel from the left). Within this pattern, higher levels of Anger were found, in both genders, in response to Sadness and Compassion films compared with the other ones (*p* < 0.05). The emotion Disgust was weakly elicited by the clips (low overall ratings) and mainly in women who showed higher levels of disgust to Fear and Erotic compared with the other clips (*p* < 0.05) (Fig 3, second panel from the left). The emotion Fear was strongly induced by the Fear clips compared with all other clip categories (Fig 3, third panel from the left) in both genders (all *p*s < 0.05). Moreover, in response to Fear clips women experienced the emotion of Fear with greater intensity than men (*p* < 0.05). Finally, in women fear was also perceived to a larger extent during Sadness and Compassion movies compared with the other pleasant-neutral films (all *p*s < 0.05). The emotion Joy was reported with greater intensity during both Erotic and Scenery compared to the other clip categories (all *p*s < 0.05), without significant difference between men and women (Fig 3, fourth panel from the left); moreover, Scenery excerpts prompted larger Joy ratings than Erotic ones, in both men and women (*p* < 0.05). Neutral clips were evaluated with the greatest Neutral emotion ratings (all *p*s < 0.05) (Fig 3, fifth panel from the left), but high Neutral ratings were attributed by women also to Scenery and Erotic clips relative to the other categories (all *p*s < 0.05). High ratings of emotion Sadness were reported to both Sadness and Compassion films, significantly more in women than in men (*p* < 0.05 for both categories). In addition, Sadness was experienced with greater intensity in response to Compassion than to Sadness clips (*p* < 0.05) (Fig 3, sixth panel from the left). Finally, even if the Surprise emotion was characterized by an overall low rating, greater levels were found in response to Fear and Scenery compared with the other films (all *p*s < 0.05) (Fig 3, last panel on the right).

### Emotional adjectives

ANOVAs on the ratings of emotional adjectives showed a significant main effect of film category for all the items, and a significant interaction Film Category*Sex for all but two items, namely Enthusiast and Enchanted (results of the F statistics are reported between parentheses).

**Fig 4:**
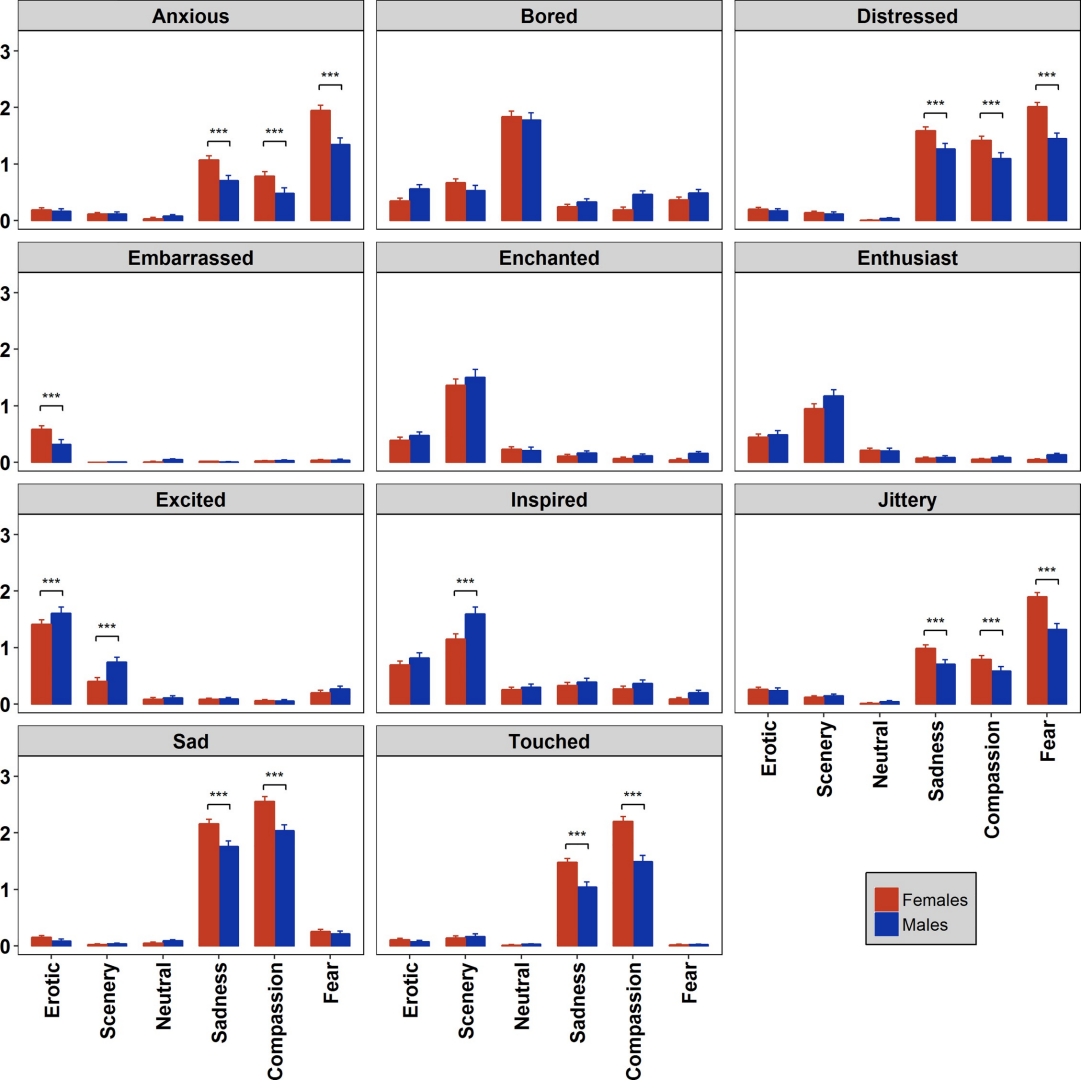
Evaluation of the six film categories according to eleven emotional adjectives. Asterisks indicate significant (p<0.05) gender differences. Bars represent SE.

The adjectives Anxious, Distressed and Jittery were naturally attributed to the three negative film categories. Indeed, statistical analysis showed that Fear clips received the highest ratings on these three items (F(5,860) = 11.11, *p*<0.0001, η^2^_p_ = 0.06, F(5,680) = 10.49, *p*<0.0001, η^2^_p_ = 0.07, and F(5,860) = 9.51, *p*<0.0001, η^2^_p_ = 0.05, respectively), with women reporting higher levels compared to men (see Fig 4). These adjectives also highlighted differences between Sadness and Compassion clips. Both males (p < 0.05) and females (p < 0.05) reported to feel more Anxious and Distressed in response to Sadness than to Compassion clips. Women also reported to feel more Jittery after presentation of Sadness compared to Compassion excerpts, while this comparison in men was marginally significant (p = 0.07).

Analysis of the item Bored showed that clips included in the Neutral category were rated as being more boring than any other clips (F(5,860) = 3.41, *p* = 0.004, η^2^_p_ = 0.02), both by men and women.

Erotic clips received significantly high rating on the item Embarrassed (F(5,860) = 7.26, *p*<0.0001, η^2^_p_ = 0.04), with female participants reporting feeling more embarrassed when watching these films than males participants.

For the adjective Excited, Erotic films were evaluated as the most exciting category. Men reported more excitement to Erotic clips than women (F(5,860) = 3.29, *p* = 0.005, η^2^_p_ = 0.02). Also Scenery clips were judged as more exciting than the remaining Film categories, again with males assigning higher scores than females.

The adjectives Enthusiast (F(5,860) = 138.38, *p*<0.0001, η^2^_p_ = 0.45), Enchanted (F(5,680) = 158.58, *p*<0.0001, η^2^_p_ = 0.54) and Inspired (F(5,860) = 3.17, *p* = 0.007, η^2^_p_ = 0.02) were clearly associated with Scenery compared with the other clips. Men were more inspired by Scenery films than women.

The adjectives Sad and Touched revealed to be more sensitive to the emotions elicited by the clips comprised in the Sadness and Compassion categories (F(5,680) = 10.24, *p*<0.0001, η^2^_p_ = 0.07 and F(5,860) = 18.37, *p*<0.0001, η^2^_p_ = 0.10, respectively) compared with the other films. Women scored higher than males. Analyses also revealed that Compassion clips received higher scores, independent from gender, namely they were described as being more sad and touching compared to the clips included in the Sadness category.

**Fig 5:**
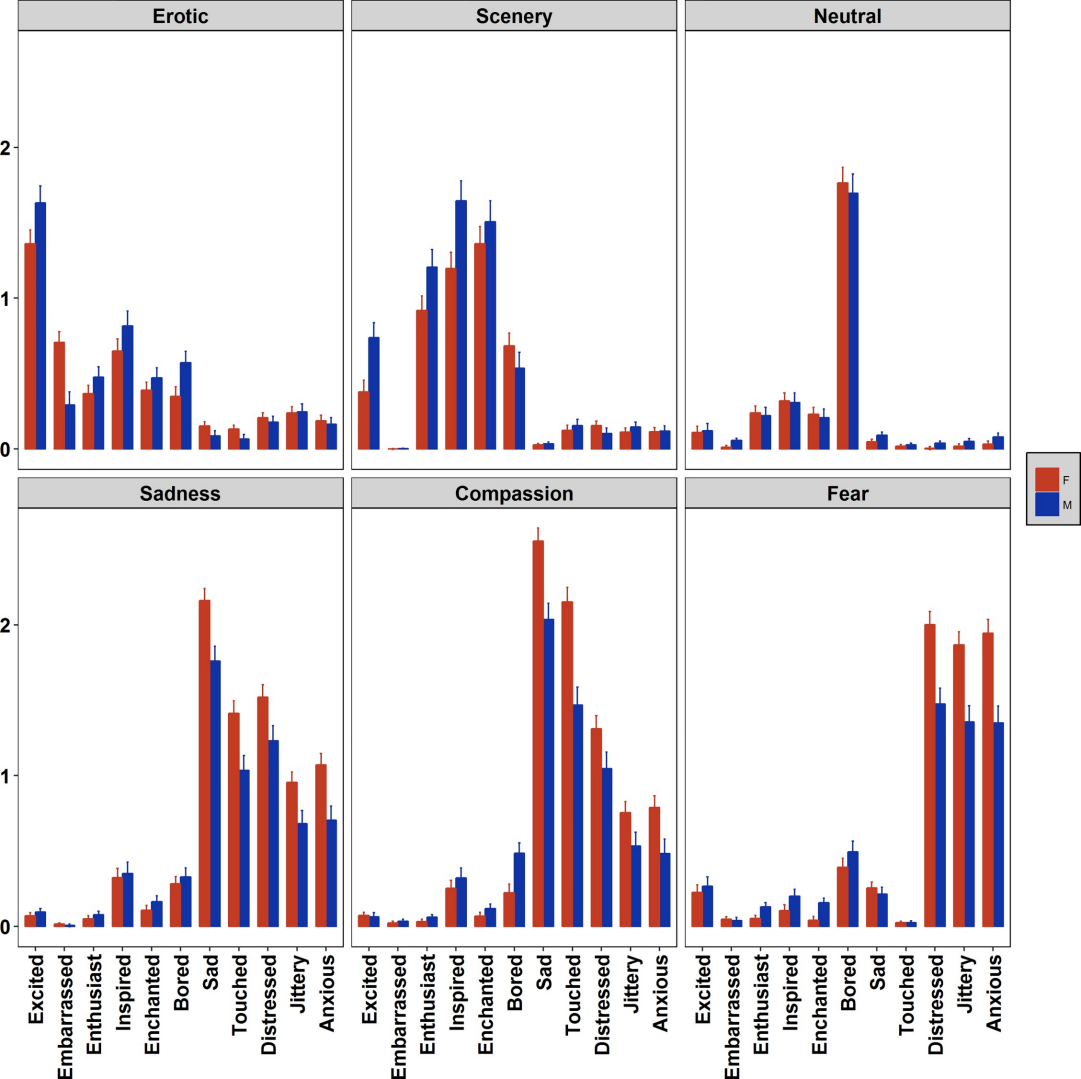
Comparison of the eleven emotional adjectives for each film clip category. Bars represent SE.

The results of the ANOVAs carried out to test, for each film category, which affective state the participants experienced with greater intensity, revealed a significant main effect for all the categories, and a significant interaction Adjective*Sex for all but one category, namely the Neutral one (results of the F statistics are reported between parentheses; see Fig 5).

For Erotic movies (F(10,1360) = 5.36, p < 0.0001, η^2^_p_ = 0.04) post-hoc pairwise comparisons revealed that Excited was the adjective which achieved the highest ratings by both males and females compared with all other adjectives (p<0.05), followed by Inspired in males and both Inspired and Embarrassed in females compared with the negative adjectives, Sad, Touched, Distressed, Jittery, Anxious (all ps< 0.05).

In response to Scenery clips (F(10,1360) = 3.53, p < 0.0001, η^2^_p_ = 0.02) participants reported highest ratings for the adjectives Inspired and Enchanted followed by the adjective Enthusiast compared to all the others adjectives (all ps< 0.05).

For the Neutral category the significant Adjective main effect (F(10,1360) = 234.5, p < 0.0001, η^2^_p_ = 0.63) revealed that the adjective Bored achieved the highest score compared to all the other adjectives (all ps< 0.05).

Participants watching the clips included in Sadness category (F(10,1360) = 3.07, p < 0.0001, η^2^_p_ = 0.04) assigned the highest ratings to the adjective Sad followed by Touched and Distressed compared with all other adjectives (all ps< 0.05). Only Females participants reported similar levels of distress and feeling touched.

For Compassion clips (F(10,1360) = 10.06, p < 0.0001, η^2^_p_ = 0.07) the analysis revealed that the adjective with the largest ratings, in both males and females, was Sad followed by Touched compared with all the other adjectives (all ps< 0.05).

Finally, for the clips included in the Fear category (F(10,1360) = 13.1, p < 0.0001, η^2^_p_ = 0.08) the analysis revealed that the adjectives Anxious, Distressed and Jittery received the highest ratings compared to all the other adjectives (all ps< 0.05), with females reporting the highest levels of these emotional states compared to males (all ps< 0.05).

### Familiarity

**Fig 6:**
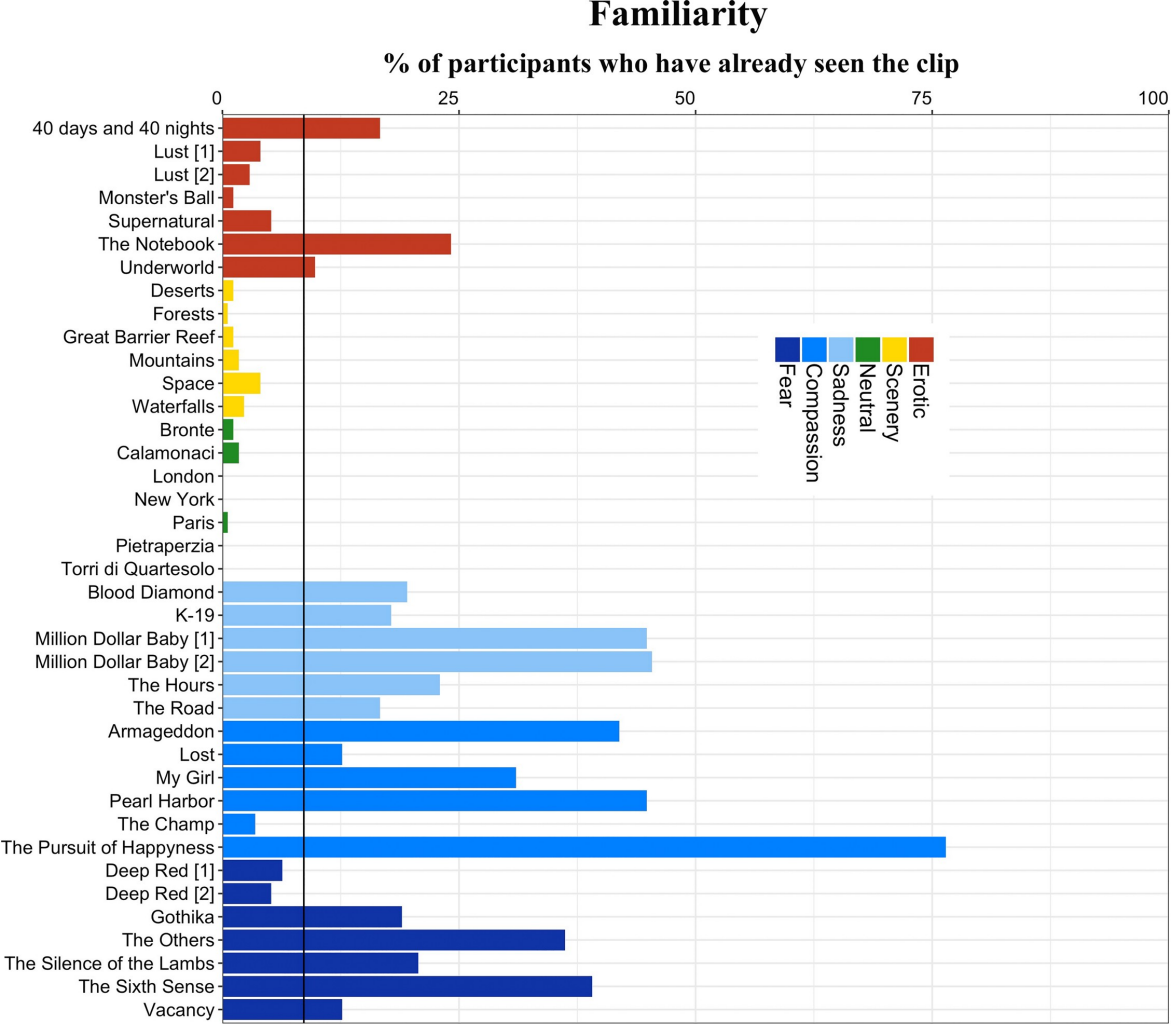
Familiarity plot. Percentage (in vertical) of participants who reported to have previously seen the clip. In horizontal axis is represented each of the 39 films. The horizontal blue line represents the threshold of 15 participants who had already watched the film (which represents 8.6% of all participants). The threshold was surpassed by 19 film clips.

Criteria for inclusion in the analysis were met by 19 clips (see Fig 6), with an average of 50.5 participants that already had seen each specific clip (min = 17 participants for *Underworld*, max = 138 for *The Pursuit of Happiness*). Concerning the impact of Familiarity on Valence ratings, the test was significant (*p* < .05) for three clips, two in the Erotic category, namely *The Notebook* (t(60) = - 2.42), *Underworld* (t(19) = -2.25) and within Fear was *The Sixth Sense* (t(122) = -2.58). In all the cases, results indicated that participants who had seen the clip previously reported larger ratings (i.e. more pleasantness) on valence scale.

Regarding arousal ratings, the test was significant (*p* < .05) for seven clips, six of which were in the Compassion or Sadness categories. The clips were *The Notebook* (t(74) = -2.51) in the Erotic category, *Blood Diamond* (t(50) = -2.58), *Armageddon* (t(168) = -3.34), *Million Dollar Baby [1]* (t(160) = -2.92), *The Pursuit of Happiness* (t(66) = -3.84), *Pearl Harbor* (t(164) = -2.14) and *K-19* (t(47) = -3.48). For all the clips, arousal perceived by participants who already had seen the clip was higher than the arousal reported by participants who did not.

## Discussion

The present study reports the validation of a new database of film excerpts for experimental emotion induction. In order to provide stimuli suitable for elicitation of either positive and negative affective state, six categories of film clips have been included, namely: Erotic, Scenery, Neutral, Sadness, Compassion, and Fear. Clips were rated on a series of emotional dimensions, assessing also the role of gender and familiarity. We predicted that Fear, Sadness and Compassion clips would have triggered an unpleasant affective state, while Erotic and Scenery clips would have elicited a positive emotional state. Analysis of valence ratings confirmed our expectation, showing that participants reported higher pleasantness in response to the latter categories of clips compared to the negative and neutral ones. In addition, Joy was the emotion reported with greater intensity in response to both Erotic and Scenery films.

These results confirm the effectiveness of Erotic excerpts to elicit in the viewer a positive emotional state, characterized by an approaching motivational tendency, extending the findings on emotional pictures research [4,51] to the field of emotional film clips. Moreover, analysis of the arousal ratings showed that the arousal elicited by erotic scenes did not differ from the one elicited by fearful excerpts. This is interesting since previous studies rarely measured the levels of arousal characterizing the emotions elicited by their clips, leaving unaddressed the impact of this variable. Results concerning the pleasantness of the affective state induced by viewing nature documentaries support our claim against the inclusion of clips with this content under the label of neutral stimuli. Participants not only judged scenes depicting natural environments as being more pleasant and more arousing compared to urban documentaries, but they also reported feeling more inspired, enthusiast and enchanted when viewing these clips. Placing Scenery in the positive group of clips in the end was consistent with our results but in past research Scenery was placed in the Neutral emotional group, thus raising some conflicting results. Indeed, Gilman and colleagues [16] included in their validation a clip extracted from a naturalistic documentary, hypothesizing that it could serve as a neutral stimulus. They found instead that, since it received moderately high ratings in positive emotionality, it did not satisfy the criteria for classifying it as affectively neutral. Carvalho and colleagues [24] also reported that scenery clips are rated as more pleasant compared to clips depicting object manipulation. The latter category was considered by authors as a true neutral condition. Our results clearly discourage the use of natural scenes as a neutral condition, and highlight their effectiveness as elicitors of positive affect. Ratings of the emotional adjectives further characterize the affective state prompted by this kind of clips, supporting their use in laboratory investigation for inducing aesthetic and contemplative emotional states, but also for studying the psychophysiological effects of the exposure to natural environments [52].

When selecting clips for the Fear category, we included only suspenseful excerpts depicting the imminence of threat and danger for the characters, excluding scenes displaying blood and/or mutilations. Although films depicting violent harm and injuries are a very effective method for eliciting fear and anxiety [12,24], blood and mutilations prompt in the viewer very specific cognitive, behavioral and physiological reactions [28,29,53], related to the concurrent disgusting nature of this kind of scenes. Our results showed that even without blood and mutilation, clips included in this category were highly effective in inducing Fear. In addition, these clips prompted the greatest levels of anxiety, jittery and distress, reflecting the thrilling nature of the selected excerpts. In line with our selection criteria, the disgust reported by participants in response to these films was very low.

In this research we also made a distinction among different kind of sadness-eliciting clips. We included film clips depicting characters crying following an important loss in the Compassion category, while clips featuring characters in loneliness, helplessness and desolation were included in the Sadness category. As expected, both these categories were effective at eliciting a negative and highly arousing affective state, whose prevalent emotion was Sadness. Interestingly, participants rated their feelings as being more sad and touched in response to Compassion clips compared to Sadness, while they felt more anxious and distressed in response to Sadness clips than to Compassion. These results support our initial claim that these categories would elicit emotional states not equivalent and only partially overlapped. Compassion clips elicited an affective state characterized by being touched and a tendency to approach the suffering characters, in line with the idea that viewing another person crying from emotional sufferance would trigger a biologically grounded automatic empathic response aimed to support the suffering person [33,54,55], which makes these stimuli suitable for investigation of empathic processing and prosocial behaviors. The observation of greater levels of sad mood induced by the Compassion compared to Sadness clips, while arousal level was equivalent, points to a biological stronger response induced by the first category. On the other hand, films included in the Sadness category were able to elicit a sad affective state which was also characterized by distress and anxiety, and this may be useful for experimental induction of depressive mood.

We also explored the impact of familiarity of stimulus material on affective reactions, aiming to replicate previous studies reporting [14,15] that familiar clips bolster the affective experience of the viewer. Due to an expectation mechanism, familiarity increases the arousal experienced during the viewing of the clips, irrespective of the positive/negative content of the stimuli. Moreover, our results showed that familiarity elicited greater pleasantness on positive clips only, while participants who watched a negative clip for the first time reported greater unpleasantness. Thus, having already watched a film tends to enhance all emotional responses: it seems that the main mechanism underlying this effect is sensitization rather than habituation. Emotions overrule cognitive mechanisms associated with habituation and this makes negative clips, especially the Fear ones, useful in paradigms resembling post-traumatic stress disorder.

Concerning gender differences in emotional responses, men and women showed a substantially similar pattern for emotional categorization of clips (Fig 3), with a few expected results: in line with sex differences found for arousal ratings, women attributed larger ratings than males in the categorization of Fear clips as fear and Sadness-Compassion clips in the sadness category. Instead, several interesting gender differences have been found for the emotional adjectives (Fig 4). After the Erotic clips, women reported feeling more embarrassed than males. This effect was not unexpected since it was previously reported [47,48], and it is related to the socially-sensitive and intimate nature of the content of these clips, to which women appear to be more susceptible. Probably this effect is strengthened by the feeling to be under investigation (although in an anonymous setting, participants were tested in groups but tests were anonymous). Men were more excited than women by the Erotic clips. They were also more aroused, excited and inspired than women after the Scenery clips. This result was unexpected and deserves further investigation. We expected women to be in general more sensitive to emotional stimulation including wilderness contents, but perhaps in men the evolutionary gender-specific tendency to explore the environment is stronger and this might be related to the fascination for wild landscapes. With regard to negative clips, women revealed to be more touched and sad than men after the Sadness and Compassion clips. Similarly, they reported also larger ratings of distress, jittery and anxiety compared with men to the three negative clips, Sadness, Compassion and Fear. This is in line with past research on sex differences in emotions using unpleasant slides [35,56], but it further specifies the differences with movies of three specific negative categories.

In conclusion, the present database revealed to be effective for affect manipulation and elicitation of different emotional states. It was built with the purpose to serve as a flexible tool for researchers, who could select the stimuli needed for their experiments adopting either a categorical or a dimensional perspective. With respect to past databases, we aimed to create new highly homogeneous categories of clips to avoid within-category mixture of contrasting/confounding emotions: Fear was not contaminated with blood-mutilation contents, Neutral did not include wilderness which instead was created as a positive independent category, Sadness was split into Sad and Compassion distinct categories. Erotic, rarely included in past databases, but extensively used as single category in many experiments, was not mixed with other positive categories. A unique feature of E-MOVIE was the introduction of two new emotional categories not present in current similar film databases: Scenery and Compassion. Interestingly, there was a gender effect with males responding more than women to Scenery and women more than men to Compassion. These new categories will allow to test novel evolutionistic hypotheses on emotions as Scenery is a positive category inducing in the observer an aesthetic and contemplative response with respect to the Erotic ones. Compassion category may serve to generate specific hypotheses on individuals with personality or psychiatric disorders characterized by altered empathy. An additional important feature was the measure of arousal levels to overcome an important limitation of some previous validations. Indeed, many results on emotion-related effects, especially in studies based on psychophysiological methods, may strongly depend on arousal levels unbalanced across different emotion categories. Thus, although it is true that different affective states are intrinsically characterized by different levels of arousal, it is also important to control this variable when differences among emotions are under investigation. One interesting feature of our sample is that all categories (excluded the Neutral) induced relative high levels of arousal. In particular, although the highest levels of arousal were reached by some Erotic and Fear clips, it is possible to select a subsample of clips having comparable arousal levels across the main five emotional categories: this allows to study emotional responses induced by different emotional categories by keeping well balanced the arousal level. As a final unique feature, our sample of clips was implemented with the aim of keeping clip duration equal across all categories. The duration was selected in the scale of 2 min which, from one side, allows the induction of most emotions, also those that take more time to develop (e.g., sadness, compassion), from the other side this duration is compatible with the needs of most neuroimaging and psychophysiological paradigms, also allowing, in one experiment, to use more clips from each emotional category to avoid film-specific response related to single long-clip presentation. Future developments of this database include: adding new homogeneous emotional categories (e.g. blood-mutilation, dirt-disgust, etc.); enlarging the sample of participants across different countries, languages and cultures; measuring the central and peripheral physiological correlates of the emotions induced by these clip categories [57]; studying neurological and psychiatric patients with specific deficits in emotional responding.

## Supporting information

S1 File Table with all E-MOVIE normative data. (XLSX)

## References

[1] Rottenberg J, Ray RD, Gross JJ. Emotion elicitation using films. Handbook of emotion elicitation and assessment. Handbook of emotion elicitation and assessment Series in affective science. 2007 p. 2–28.

[2] Bradley MM, Lang PJ. The International Affective Digitized Sounds (2nd Edition; IADS-2): Affective ratings of sounds and instruction manual Gainesville, FL: University of Florida; 2007.

[3] Felnhofer A, Kothgassner OD, Schmidt M, Heinzle AK, Beutl L, Hlavacs H, et al Is virtual reality emotionally arousing? Investigating five emotion inducing virtual park scenarios. Int J Hum Comput Stud. 2015;82:48–56.

[4] Lang PJ, Bradley MM, Cuthbert BN. International affective picture system (IAPS): Instruction manual and affective ratings. Technical Report A-2. Gainesville, FL: University of Florida; 1999.

[5] Lang PJ, Bradley MM, Cuthbert BN. International affective picture system (IAPS): Instruction manual and affective ratings. Technical Report A-2. Gainesville, FL: University of Florida; 1999.

[6] Zentner M, Grandjean D, Scherer KR. Emotions evoked by the sound of music: characterization, classification, and measurement. Emotion. 2008;8(4):494–521. 10.1037/1528-3542.8.4.494 18729581

[7] De Cesarei A, Loftus GR, Mastria S, Codispoti M. Understanding natural scenes: Contributions of image statistics. Neurosci Biobehav Rev. 2017;74:44–57. 10.1016/j.neubiorev.2017.01.012 28089884

[8] Ekman P, Friesen W. Pictures of Facial Affect Palo Alto (CA): Consulting Psychology Press; 1976.

[9] Lundqvist D, Flykt A, Öhman A. The Karolinska Directed Emotional Faces—KDEF, CD ROM from Department of Clinical Neuroscience, Psychology section Karolinska Institut, ISBN 91-630-7164-9. 1998;

[10] Marchewka A, Zurawski L, Jednoróg K, Grabowska A. The Nencki Affective Picture System (NAPS): Introduction to a novel, standardized, wide-range, high-quality, realistic picture database. Behav Res Methods. 2014;46(2):596–610. 10.3758/s13428-013-0379-1 23996831 PMC4030128

[11] Westermann R, Stahl G, Hesse FW. Relative effectiveness and validity of mood induction procedures: a meta-analysis. Eur J Soc Psychol. 1996;26:557–80.

[12] Schaefer A, Nils F, Sanchez X, Philippot P. Assessing the effectiveness of a large database of emotion-eliciting films: A new tool for emotion researchers. Cogn Emot. 2010;24(7):1153–72.

[13] Philippot P. Inducing and assessing differentiated emotion-feeling states in the laboratory. Cogn Emot. 1993 3 7;7(2):171–93. 10.1080/02699939308409183 27102736

[14] Gross JJ, Levenson RW. Emotion elicitation using films. Cogn Emot. 1995 1;9(1):87–108.

[15] Gabert-Quillen CA, Bartolini EE, Abravanel BT, Sanislow CA. Ratings for emotion film clips. Behav Res Methods. 2015;47(3):773–87. 10.3758/s13428-014-0500-0 24984981 PMC6445277

[16] Gilman TL, Shaheen R, Nylocks KM, Halachoff D, Chapman J, Flynn JJ, et al A film set for the elicitation of emotion in research: A comprehensive catalog derived from four decades of investigation. Behav Res Methods. 2017 1 11;1–22. 10.3758/s13428-015-0685-x 28078572

[17] Jenkins LM, Andrewes DG. A New Set of Standardised Verbal and Non-verbal Contemporary Film Stimuli for the Elicitation of Emotions. Brain Impair. 2012;13(2):212–27.

[18] Samson AC, Kreibig SD, Soderstrom B, Wade AA, Gross JJ. Eliciting positive, negative and mixed emotional states: A film library for affective scientists. Cogn Emot. 2015;30(5):827–56. 10.1080/02699931.2015.1031089 25929696

[19] von Leupoldt A, Rohde J, Beregova A, Thordsen-Sörensen I, zur Nieden J, Dahme B. Films for eliciting emotional states in children. Behav Res Methods. 2007;39(3):606–9. 17958174 10.3758/bf03193032

[20] Sternbach RA. Assessing differential autonomic patterns in emotions. J Psychosom Res. 1962;6(2):87–91. 13917003 10.1016/0022-3999(62)90059-4

[21] Kreibig SD. Autonomic nervous system activity in emotion: A review. Biol Psychol. 2010;84:14–41 10.1016/j.biopsycho.2010.03.010 20371374

[22] Golland Y, Keissar K, Levit‐Binnun N. Studying the dynamics of autonomic activity during emotional experience. Psychophysiology. 2014;51(11):1101–1111. 10.1111/psyp.12261 25039415

[23] Golland Y, Hakim A, Aloni T, Schaefer S, Levit-Binnun N. Affect dynamics of facial EMG during continuous emotional experiences. Biol Psychol. 2018;139:47–58. 10.1016/j.biopsycho.2018.10.003 30300673

[24] Carvalho S, Leite J, Galdo-Álvarez S, Gonçalves ÓF. The emotional movie database (EMDB): A self-report and psychophysiological study. Appl Psychophysiol Biofeedback. 2012;37(4):279–94. 10.1007/s10484-012-9201-6 22767079

[25] Hewig J, Hagemann D, Seifert J, Gollwitzer M, Naumann E, Bartussek D. A revised film set for the induction of basic emotions. Cogn Emot. 2005;19(7):1095–109.

[26] Frijda NH. Aesthetic emotions and reality. Am Psychol. 1989;44(12):1546–7.

[27] Ekman P. An argument for basic emotions. Cogn Emot. 1992;6(3):169–200.

[28] Palomba D, Sarlo M, Angrilli A, Mini A, Stegagno L. Cardiac responses associated with affective processing of unpleasant film stimuli. Int J Psychophysiol. 2000;36(1):45–57. 10.1016/s0167-8760(99)00099-9 10700622

[29] Sarlo M, Buodo G, Poli S, Palomba D. Changes in EEG alpha power to different disgust elicitors: The specificity of mutilations. Neurosci Lett. 2005;382(3):291–6. 10.1016/j.neulet.2005.03.037 15925105

[30] Gross JJ, Fredrickson BL, Levenson RW. The psychophysiology of crying. Psychophysiology. 1994;31(5):460–8. 10.1111/j.1469-8986.1994.tb01049.x 7972600

[31] Hendriks MCP, Croon M a, Vingerhoets AJJM. Social reactions to adult crying: the help-soliciting function of tears. J Soc Psychol. 2008;148(1):22–41. 10.3200/SOCP.148.1.22-42 18476481

[32] Hendriks MCP, Vingerhoets AJJM. Social messages of crying faces: Their influence on anticipated person perception, emotions and behavioural responses. Cogn Emot. 2006;20(6):878–86.

[33] Decety J, Meyer M. From emotion resonance to empathic understanding: A social developmental neuroscience account. Dev Psychopathol. 2008 9 7;20(04):1053. 18838031 10.1017/S0954579408000503

[34] Singer T, Klimecki OM. Empathy and compassion. Curr Biol. 2014;24 (18):875–878. 25247366 10.1016/j.cub.2014.06.054

[35] Bianchin M, Angrilli A. Gender differences in emotional responses: A psychophysiological study. Physiol Behav. 2012;105(4):925–32. 10.1016/j.physbeh.2011.10.031 22108508

[36] Bradley MM, Codispoti M, Cuthbert BN, Lang PJ. Emotion and motivation I: defensive and appetitive reactions in picture processing. Emotion. 2001 9;1(3):276–98. 12934687

[37] Codispoti M, Surcinelli P, Baldaro B. Watching emotional movies: Affective reactions and gender differences. Int J Psychophysiol. 2008;69(2):90–5. 10.1016/j.ijpsycho.2008.03.004 18433903

[38] Herbert BM, Pollatos O, Schandry R. Interoceptive sensitivity and emotion processing: An EEG study. Int J Psychophysiol. 2007;65(3):214–27. 17543405 10.1016/j.ijpsycho.2007.04.007

[39] Karama S, Armony J, Beauregard M. Film excerpts shown to specifically elicit various affects lead to overlapping activation foci in a large set of symmetrical brain regions in males. PLoS One. 2011;6(7). 10.1371/journal.pone.0022343 PMC3144904 21818311

[40] Stevens JS, Hamann S. Sex differences in brain activation to emotional stimuli: A meta-analysis of neuroimaging studies. Neuropsychologia. 2012;50(7):1578–93. 10.1016/j.neuropsychologia.2012.03.011 22450197

[41] Wierzba M, Riegel M, Pucz A, Leśniewska Z, Dragan WŁ, Gola M, et al Erotic subset for the Nencki Affective Picture System (NAPS ERO): cross-sexual comparison study. Front Psychol. 2015;6:1336 10.3389/fpsyg.2015.01336 26441715 PMC4564755

[42] Hartig T, Evans GW, Jamner LD, Davis DS, Gärling T. Tracking restoration in natural and urban field settings. J Environ Psychol. 2003;23(2):109–23.

[43] Johnsen SÅK, Rydstedt LW, Rydstedt LW. Active Use of the Natural Environment for Emotion Regulation. Eur J Psychol. 2013 11 29;9(4):798–819.

[44] Kaplan S. The restorative benefits of nature: Toward an integrative framework. J Environ Psychol. 1995 9;15(3):169–82.

[45] Ulrich RS, Simons RF, Losito BD, Fiorito E, Miles MA, Zelson M. Stress recovery during exposure to natural and urban environments. J Environ Psychol. 1991 9;11(3):201–30.

[46] van den Berg AE, Koole SL, van der Wulp NY. Environmental preference and restoration: (How) are they related? J Environ Psychol. 2003;23(2):135–46.

[47] Costa M, Dinsbach W, Manstead ASR, Ricci Bitti PE. Social presence, embarrassment, and nonverbal behavior. J Nonverbal Behav. 2001;25(4):225–40.

[48] Maffei A, Vencato V, Angrilli A. Sex differences in emotional evaluation of film clips: Interaction with five high arousal emotional categories. PLoS One. 2015;10(12). 10.1371/journal.pone.0145562 PMC4696842 26717488

[49] Bradley MM, Lang PJ. Measuring emotion: The self-assessment manikin and the semantic differential. J Behav Ther Exp Psychiatry. 1994;25(1):49–59. 7962581 10.1016/0005-7916(94)90063-9

[50] Welch BL. The generalization of `Student’s’ problem when several different population variances are involved. Biometrika. 1947;34(1/2):28–35. 20287819 10.1093/biomet/34.1-2.28

[51] Lang PJ, Greenwald MK, Bradley MM, Hamm AO. Looking at pictures: affective, facial, visceral, and behavioral reactions. Psychophysiology. 1993;30(3):261–73. 10.1111/j.1469-8986.1993.tb03352.x 8497555

[52] Beute F, de Kort YAW. Salutogenic effects of the environment: Review of health protective effects of nature and daylight. Appl Psychol Heal Well-Being. 2014;6(1):67–95. 10.1111/aphw.12019 24259414

[53] Cisler JM, Olatunji BO, Lohr JM, Williams NL. Attentional bias differences between fear and disgust: Implications for the role of disgust in disgust-related anxiety disorders. Cogn Emot. 2009 6;23(4):675–87. 10.1080/02699930802051599 20589224 PMC2892866

[54] Goetz J, Keltner D, Simon-Thomas E. Compassion: an evolutionary analysis and empirical review. Psychol Bull. 2010;136(3):351–74. 10.1037/a0018807 20438142 PMC2864937

[55] Menninghaus W, Wagner V, Hanich J, Wassiliwizky E, Kuehnast M, Jacobsen T. Towards a psychological construct of being moved. PLoS One. 2015 1 4;10(6):e0128451 10.1371/journal.pone.0128451 26042816 PMC4456364

[56] Bradley MM, Codispoti M, Sabatinelli D, Lang PJ. Emotion and motivation II: sex differences in picture processing. Emotion. 2001;1(3):300–19. 12934688

[57] Maffei A, Angrilli A. Spontaneous blink rate as an index of attention and emotion during film clips viewing. Physiol Behav. 2019;204(2):256–63. 10.1016/j.physbeh.2019.02.037 30822434

